# RNAlyzer—novel approach for quality analysis of RNA structural models

**DOI:** 10.1093/nar/gkt318

**Published:** 2013-04-25

**Authors:** Piotr Lukasiak, Maciej Antczak, Tomasz Ratajczak, Janusz M. Bujnicki, Marta Szachniuk, Ryszard W. Adamiak, Mariusz Popenda, Jacek Blazewicz

**Affiliations:** ^1^Institute of Computing Science, Poznan University of Technology, 60-965 Poznan, Poland, ^2^Institute of Bioorganic Chemistry, Polish Academy of Sciences, 61-704 Poznan, Poland, ^3^Laboratory of Bioinformatics and Protein Engineering, International Institute of Molecular and Cell Biology, 02-109 Warsaw, Poland and ^4^Laboratory of Bioinformatics, Institute of Molecular Biology and Biotechnology, Adam Mickiewicz University, 61-614 Poznan, Poland

## Abstract

The continuously increasing amount of RNA sequence and experimentally determined 3D structure data drives the development of computational methods supporting exploration of these data. Contemporary functional analysis of RNA molecules, such as ribozymes or riboswitches, covers various issues, among which tertiary structure modeling becomes more and more important. A growing number of tools to model and predict RNA structure calls for an evaluation of these tools and the quality of outcomes their produce. Thus, the development of reliable methods designed to meet this need is relevant in the context of RNA tertiary structure analysis and can highly influence the quality and usefulness of RNA tertiary structure prediction in the nearest future. Here, we present RNAlyzer—a computational method for comparison of RNA 3D models with the reference structure and for discrimination between the correct and incorrect models. Our approach is based on the idea of local neighborhood, defined as a set of atoms included in the sphere centered around a user-defined atom. A unique feature of the RNAlyzer is the simultaneous visualization of the model-reference structure distance at different levels of detail, from the individual residues to the entire molecules.

## INTRODUCTION

RNA is one of the leading actors in all life processes. Its catalytic prowess, biological importance and ability to form complex structures have made this molecule an important subject of research in recent years. A variety of biological functions carried out by RNAs comes, inter alia, from their three dimensional structures. Thus, determination of the RNA structure is an important step in RNA analysis (1,2). Unfortunately, experimental determination of the RNA structure is difficult and time-consuming. The most common ways to obtain high-resolution models of the RNA 3D structure are based on experimental methods such as X-ray crystallography and nuclear magnetic resonance (NMR) spectroscopy, but these methods cannot be applied directly to all RNAs (3). An alternative to experimental determination is the computational modeling of the RNA 3D structure, often based on spatial restraints derived from low-resolution experimental data other than those obtained by crystallography or NMR. Computational approaches to RNA modeling include e.g. conformational space searching (4–7), interactive modeling (8–11) and comparative modeling (12,13).

The growing number of RNA sequences and experimentally determined 3D structure data has prompted the development of new methods and software for RNA modeling (14–20). Only recently a benchmarking experiment has been organized to compare the reported methods on a common set of prediction targets and with common measures (21).

The assessment of the applicability of RNA models requires the development of reliable computational methods to compare them with the experimentally determined structures and thereby evaluate the accuracy of predictions. Correct identification of accurate models and recognition of methods that are likely to generate accurate models is an important aspect of model evaluation. Several metrics are currently used to evaluate 3D models of macromolecules. The most common metric to describe the mutual global deviation of two structures is the root mean square deviation (RMSD) calculated for pairs of corresponding atoms, following the optimal superposition of the two compared structures. Another common metric is the global distance test (GDT) originally invented for the comparison of protein structures (22,23). Metrics designed specifically for RNA include the deformation index (DI) and interaction network fidelity (INF), both of which take into account base–base interactions within the structures, and the deformation profile (DP), which takes into account both global and local differences (3).

In this article, we present a new computational method designed to support comparison of RNA structure models to a reference structure. The method is able to discriminate between correct and incorrect predictions in entire spectrum of detail (from local to global perspective). Our method is equipped with an advanced visualization function, and allows the user to compare tertiary structures simultaneously at different levels of accuracy.

## MATERIALS AND METHODS

**Figure 1.**
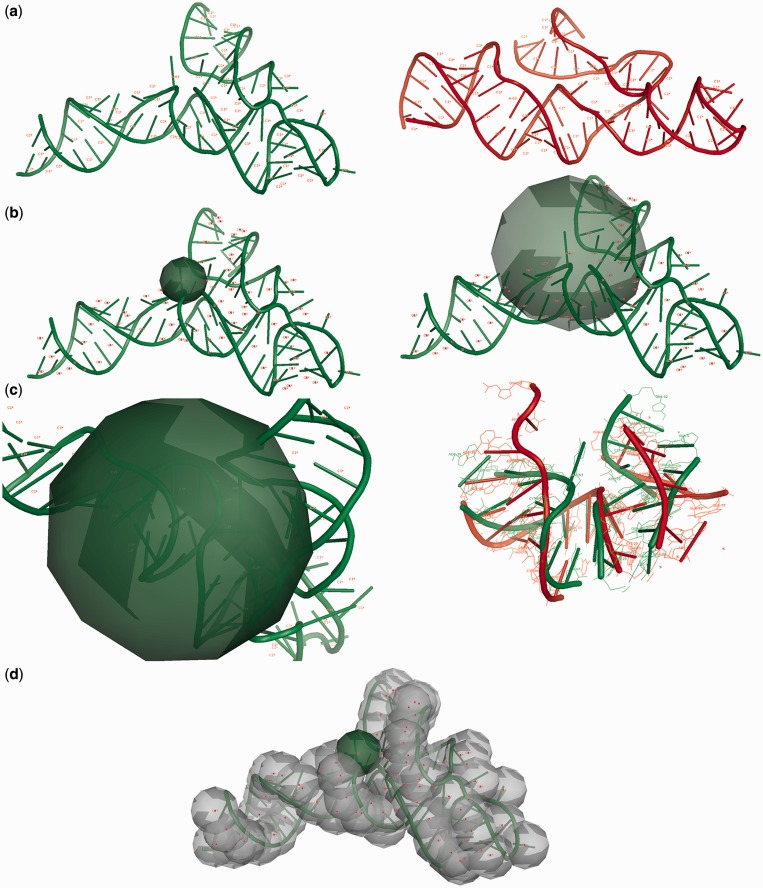
(**a**) Input data defined by the user: the reference structure (green), the model (red), the center atom of the sphere, vector of radii, level of accuracy—positions of all atoms or only the selected atom type of a particular nucleotide will be considered. (**b**) The sphere is built based on a selected atom of every residue from the reference structure; the radius can be set by the user (e.g. 6 Å—left, 18 Å—right picture). (**c**) The reference structure atoms for the selected sphere are recognized, corresponding atoms from the model are identified, both sets are superimposed, and RMSD is calculated. (**d**) Spheres are built based on a selected type of atom for every nucleotide in the reference structure, and the process from (c) is repeated (reference structure: RNA Puzzles Problem 3, model: Dokholyan_model_2 (23)).

RNAlyzer allows the user to evaluate the agreement between a model of RNA molecule and a reference structure by comparisons between sets of atoms located inside a series of spheres. A sphere with a strictly defined radius is built around an atom selected by the user (C1*, O5’, O3’ or P), which represents the center of the sphere of every nucleotide that is part of the reference structure. As a result of the sphere-building process, a set of all atoms included in a particular sphere from reference structure is obtained (no atoms from reference structure are omitted). In the next stage, for every sphere built on a particular atom belonging to nucleotide residue from the reference structure, a corresponding set of atoms from the analyzed model is identified. The alignment is done by matching atom numbers from reference and structure and model. Finally, substructures inside corresponding spheres are superimposed, and the RMSD between corresponding superimposed sets is calculated. Kabsch and McLachlan techniques (24,25) have been combined to optimally superimpose corresponding atoms sets between the reference structure and the model analyzed. The ability to select different radii allows the user to compare models at different levels of structural detail. If the sphere radius is small, then the model is analyzed from a local point of view (higher details level). Otherwise, the analysis is conducted from a global point of view (lower level of detail). If the sphere radius is equal to or greater than the radius of the whole molecule, the RMSD computed for each nucleotide value is equal to the global RMSD calculated for whole structure. Our software also allows the user to define a vector of sphere radii that can be visualized simultaneously in one plot. This information can give the overview of the model quality simultaneously for different levels of detail, from local to global perspective (Figure 1).

### Computational module

This module is responsible for checking the correctness of the input file and reporting all inconsistencies (e.g. violations of the PDB file format) to the user. Moreover, it contains functions to compute the RMSD between the model and the reference structure (for every sphere radius identified in input radii vector) based on atoms taken from spheres with different radii. The user can adjust the analysis and set initial values of several input parameters, such as a vector of radii or a type of atom that is positioned in the center of the sphere. The user can also decide whether all atoms of each particular nucleotide or only the central atom in a sphere will be considered. Following the calculations at this stage, the user can save results or proceed with visualization.

### Visualization module

The visualization module includes a tool that enables generation of five types of plots that represent different views focusing on different levels of detail. The current version of RNAlyzer is based on third-party secondary structure annotation tools such as RNAView or Annotate3D (11,15,26,27). To use the Annotate3D, the internet connection is needed. By default, the system uses RNAView to calculate and present to the user the secondary structure for the models and the reference structure.

### Data and implementation

**Figure 2.**
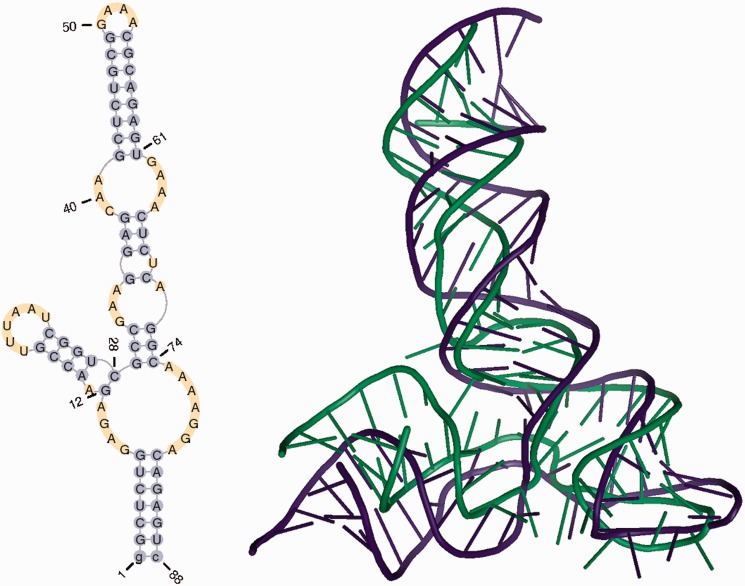
Secondary and tertiary structure of a reference molecule for Problem_3 from the RNAPuzzles challenge (23).

The plots presented below were generated for models submitted to the RNA Puzzles competition by three groups (Chen, Major and Das) (21). These groups proposed several models for RNA under Problem 3 (a riboswitch domain) (28) (Figure 2). In our analysis, we used one model for each group Chen_model_1, Major_model_2 and Das_model_3.

RNAlyzer is a standalone program written in JAVA programming language, and uses Java3D and Jmol libraries. The software runs on any modern Windows or UNIX system (including Mac OS X). RNAlyzer is free of charge for academic and commercial users. Executable version of RNAlyzer is available at http:\\rnalyzer.cs.put.poznan.pl.

#### Multi-model plot

**Figure 3.**
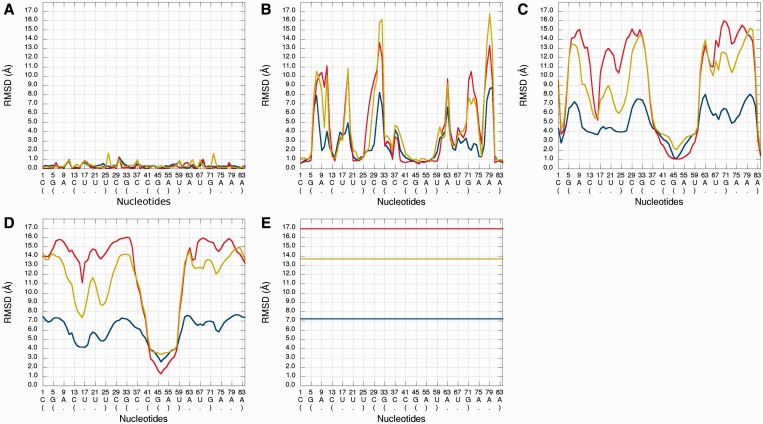
*Multi-model plot* for Problem_3; each plot (**A–E**) represents results for a different sphere radius (3, 8, 20, 38 and 300 Å). X-axis represents the order of nucleotides in the sequence, Y-axis represents the RMSD.

An example of *Multi-model plot* is presented in Figure 3. This is a multiple models curve plot, where each curve describes a single model. Each model is represented by a different color. The RMSD values between superimposed atoms from the reference structure and corresponding atoms from the analyzed model are calculated. Considered atoms are located in the sphere that is built around a selected atom (predefined by the user) for every nucleotide of the reference structure.

The *Multi-model plot* visualizes how accurate is the prediction of atoms located in the neighborhood of every nucleotide, taking into consideration current accuracy level (ie. sphere radius). Each line describes exactly one structural model—different colors correspond to different models. With a low radius (represents local perspective—high precision of assessment), the quality of local structural neighborhood of analyzed models is correct for almost all nucleotide residues of the model, because accurate modeling of chemical structures of nucleotide residues is relatively easy. With the increasing value of radius, we can easily identify the parts of models that exhibit different accuracies of prediction, from low values of RMSD that indicate correct predictions, to important structural errors (e.g. different torsion angles for particular nucleotide can cause high values of RMSD computed for its local neighborhood) that should be widely and carefully analyzed and refined. If the sphere radius reached the radius of the whole molecule, then the RMSD computed for each nucleotide is equal to the global RMSD computed for a whole structure (Figure 3).

The main feature of the *Multi-model plot* is Y-axis scaling of all predicted models to the potentially worst model, because its RMSD values are the highest. Visualization of two prediction models that differ significantly (one model—very good quality of prediction, second model—significant structural errors) on the *Multi-model* plot can be confusing because the RMSD value for the worse model may dominate the visualization of errors for the model with a better accuracy. Hence, the user can analyze each model separately.

#### RMSD averaged plot

**Figure 4.**
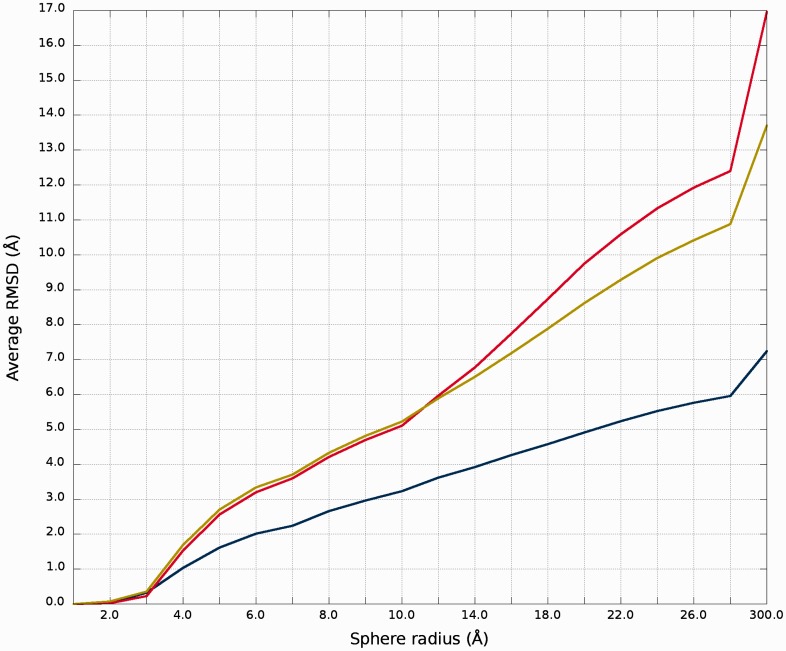
*RMSD averaged plot*; X-axis represents the sphere radius, Y-axis represents averaged RMSD for all spheres with fixed radius (different colors correspond to different models).

An example of *RMSD averaged plot* is shown in Figure 4. This is also a multiple model curve plot, where each line describes exactly single structural model (different colors correspond to different models). The difference between *Multi-model plot* and *RMSD averaged plot* is that in the latter case, the Y-axis represents values of RMSD calculated as the averaged sum of RMSD of spheres built with fixed radius for all nucleotides of the analyzed RNA molecule. This plot visualizes how averaged RMSD values change with an increasing sphere radius (ie. decreasing accuracy level). In general, this plot describes the changes of quality of prediction in entire spectrum of accuracy levels from local to global point of view.

#### 2D map plot

**Figure 5.**
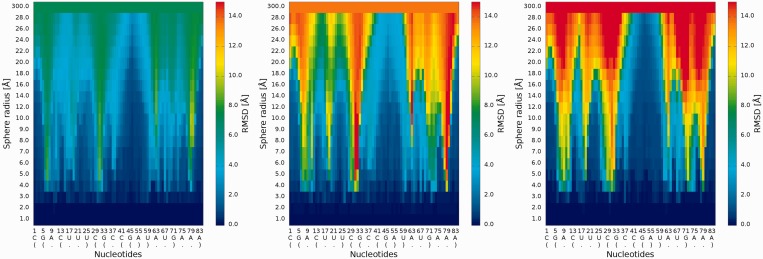
*2D map* plot—each map corresponds to exactly one of the analyzed models (left—Chen_model_1, center—Major_model_2 and right—Das_model_3); X-axis represents the sequential order of nucleotides; Y-axis represents the sphere radius; color of the cell represents the RMSD value, following the scale presented at the bottom (blue—low RMSD and high prediction quality, red—high RMSD and low prediction quality).

An example of a *2D map plot* is presented in Figure 5. This plot visualizes exactly one model at a time. The values of RMSD are visualized as a color in a spectrum between blue (high prediction quality) and red (low prediction quality). The X-axis represents residue numbers (in sequential order), and the Y-axis represents the radius values from the *spheres radii vector* defined by the user. This plot shows where the prediction is inaccurate and allows the user to identify if prediction errors are similar for different analyzed models.

#### 3D plot

**Figure 6.**
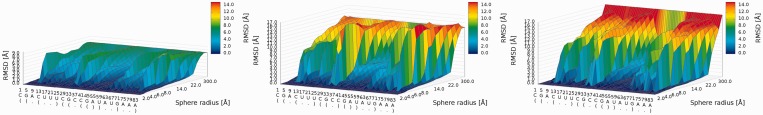
*3D plot*—analysis of three models (left—Chen_model_1, center—Major_model_2 and right—Das_model_3); X-axis represents the sequential order of nucleotides; Y-axis represents sphere radius; Z-axis represents RMSD.

An example of a *3D plot* is presented in Figure 6. The X-axis represents nucleotide numbers in sequential order; the Z-axis represents the radius values from the *spheres radii vector* defined by the user; and the Y-axis represents the RMSD values. The value of RMSD is also presented with the colored scale. Both plots (*2D map plot* and *3D plot*) describe the accuracy of structural fragments of a predicted model around certain nucleotides.

#### Cutoff plot

**Figure 7.**
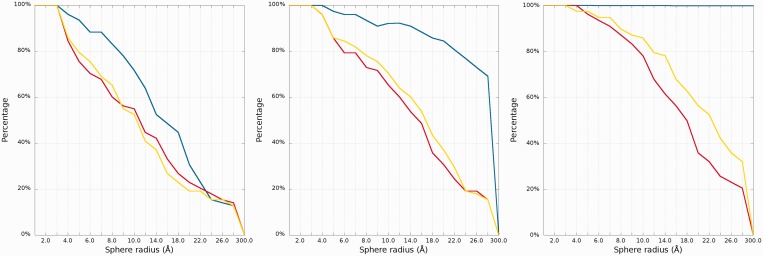
*Cutoff* plot presents a percentage of the atoms sets included in spheres with fixed radius for each considered model, which are below a selected cutoff threshold. Every curve corresponds to single model. X-axis represents sphere radius; Y-axis corresponds to percentage value. On the picture above we observe see plots for different precision values (from left to the right: 4 Å, 7 Å and 10 Å) for radius of the sphere equal to 24 Å (each predicted model is represented by a different color).

An example of a *Cutoff* plot is presented in Figure 7. *Cutoff* plot shows how accurate is the prediction of a particular model from a local point of view or, in other words, which part of the model structure is predicted correctly. The calculation is performed based on the fixed precision value (cutoff threshold) for each sphere radius, defined by the user in the spheres radii vector. As a result, the user receives information about the percentage of nucleotides predicted with the quality below specified cutoff (%). The value of cutoff threshold can be changed interactively by the user.

**Figure 8.**
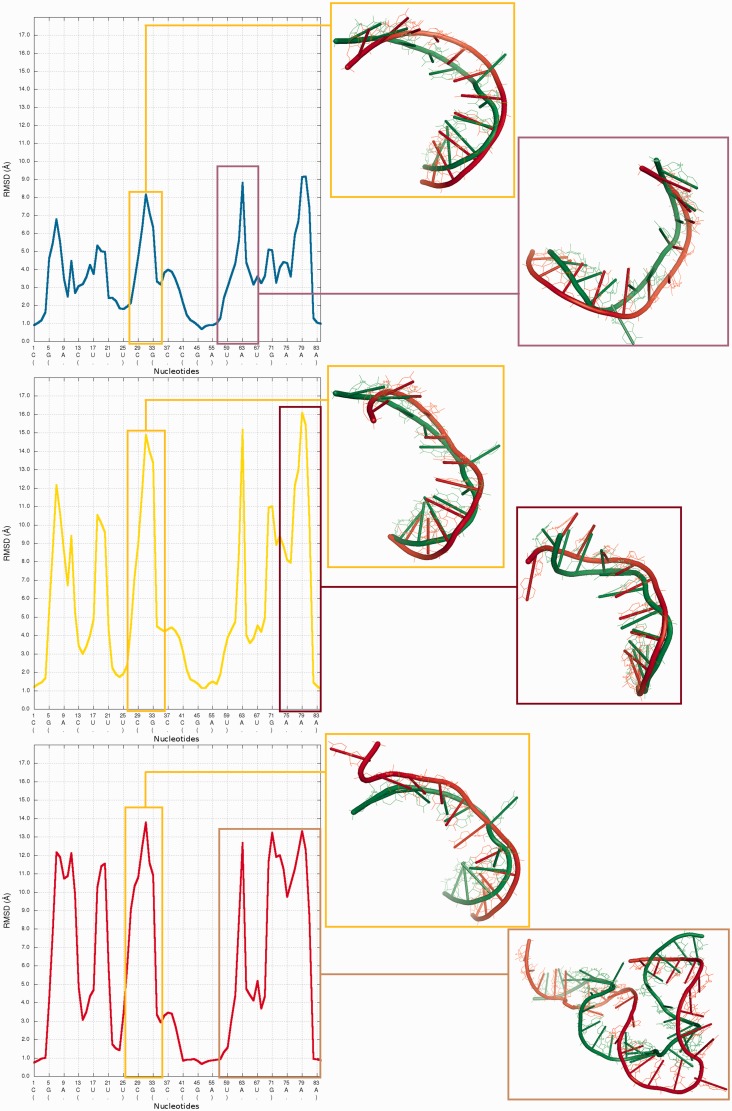
Visualization of structural fragments where the prediction model is inconsistent with the reference structure (from the top: Chen_model_1, Major_model_2 and Das_model_3). From left to right: multiple model 1D plot, 3D structure (reference structure is presented in green; model is presented in red).

Figure 8 presents structural fragments where possible prediction errors can be found in structures of models presented in Figures 3–7.

## RESULTS AND DISCUSSION

To show the advantages of the presented approach, we analyzed models of three different RNA molecules, submitted by eight research groups within the first ‘RNA Puzzles’ challenge that took place in November 2010 (21). We downloaded models from the web page of RNA Puzzles (http://paradise-ibmc.u-strasbg.fr/rnapuzzles/problems_past.html), compared them with the experimentally determined structures and analyzed the results in the context of model evaluation carried out within the RNA Puzzles experiment. The goal of the analysis presented below is to show benefits using RNAlyzer during quality evaluation process, but not to reevaluate results of RNA Puzzles.

### Problem 1 / Challenge case 1

**Figure 9.**
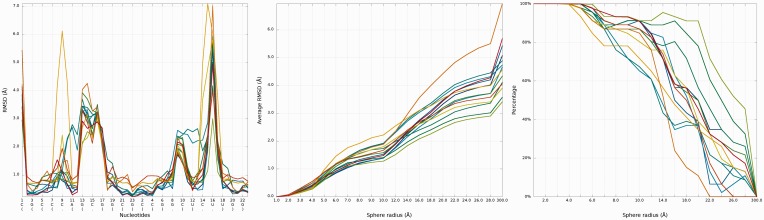
Problem 1—*Multi-model plot* (left)—prediction errors for all models are indicated in two regions. *RMSD averaged plot* (center)—for low sphere radius Bujnicki_model_1 is the best; different colors correspond to different models. *Cutoff* (right) plot shows impressive local accuracy of Das_model_3 (precision 4 Å, sphere radius 6 Å); different colors correspond to different models.

The crystal structure of the regulatory element from human thymidylate synthase mRNA (29) revealed a dimer of identical sequences, with two asymmetrical internal loops. We analyzed all fourteen models submitted for this reference structure with the RNAlyzer, using the *Multi-model plot* (Figure 9). The analysis revealed that while all the models exhibit an approximately correct global structure, they show local errors in two positions (around residue 15 and 38), which is particularly evident in the plots for the sphere radius equal to 5 Å. For Das_model_4 and Das_model_5 models, wrong local prediction is clearly visible in comparison with other models.

Looking at *RMSD averaged plot*, we can observe that local prediction in local neighborhood (up to 7 Å) is the best for Bujnicki_model_1 and Bujnicki_model_3 models (Figure 9). Analysis of *Cutoff* plot (Figure 9) illustrates that local structural inconsistencies identified in Das_model_3 are compensated by an impressive quality of predictions for other regions, the percentage of spheres below cutoff threshold growing rapidly with the increasing value of radius.

**Figure 10.**
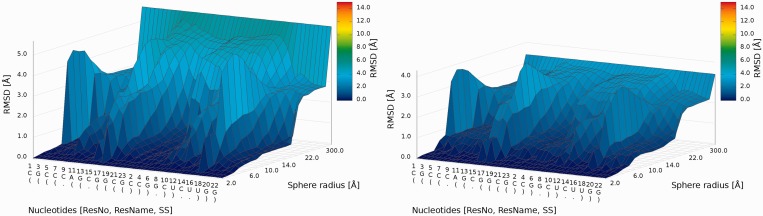
*3D* plot of Santalucia_model_1 (right) and Das_model_3 (left).

*3D plot*s indicate structural differences between both predictions (Figure 10). Das_model_3 is potentially the best predicted model. Santalucia_model_1 has some missing atoms in regions that were generally difficult to model.

**Figure 11.**
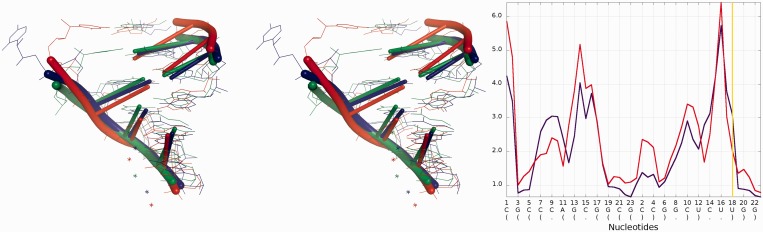
Superposition of Das_model_4 and Dokholyan_model_1 (left) with the reference structure; *Multi-model plot* (right) corresponds to discussed regions (green color—reference structure, red color—globally less accurate model (Dokholyan_model_1), blue color—globally more accurate model (Das_model_4)).

A detailed analysis of submitted models with RNAlyzer shows that this program can vividly point out to local conformations that were predicted much more accurately in globally less accurate models than their counterparts in globally more accurate models. This can be illustrated by e.g. comparison of Das_model_4 and Dokholyan_model_1 models (Figure 11). The difference of RMSD for local neighborhood around nucleotide No 18 between analyzed models is over 1 Å (for the structural motif from Dokholyan_model_1 lower RMSD was calculated), but globally the RMSD value for the Das_model_4 is around 3 Å lower than the Dokholyan_model_1.

Similar situation as presented above identified for Problem 1 is presented in Supplementary Figure S1.

### Problem 2 / Challenge case 2

**Figure 12.**
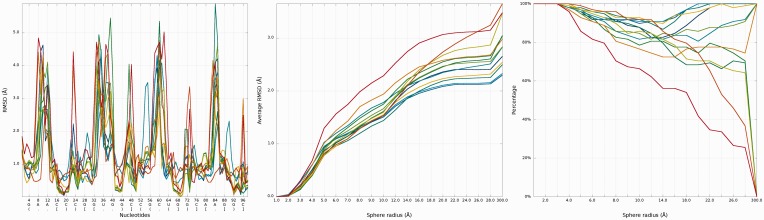
Problem 2—*Multiple 1D* plot (left)—prediction errors for all groups are indicated in several parts of the structure. *RMSD averaged plot* (center)—the Dokholyan_model_1 is the best for low sphere radius; different colors correspond to different models. Cutoff (right) plot shows impressive local prediction of Das_model_1 (precision 3 Å, sphere radius 7 Å); different colors correspond to different models.

**Figure 13.**
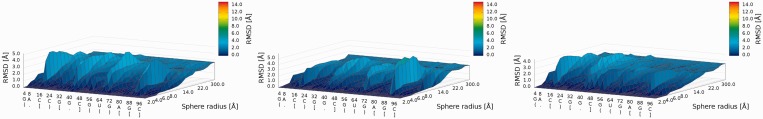
*3D plot*s of Bujnicki_model_1 (left), Das_model_1 (center) and Dokholyan_model_1 (right).

The reference molecule submitted for this challenge includes eight chains forming a square-like structure (30). The conformation of four shorter chains was provided, along with secondary structure, and the most important task was to model the remaining four chains, in particular loop regions. We analyzed 12 models submitted is response to this challenge. The input RNA molecule is larger than RNA molecule from Problem 1, so the total number of local structural errors is also much larger, but the impact of these errors on the global accuracy of the models is smaller, most likely owing to the constrains of the starting structure (Figure 12). The average of local RMSD computed for low values of sphere radius gives advantage to Dokholyan_model_1, but with increasing radius of the sphere the Bujnicki_model_2 and Bujnicki_model_3 prove to be the best. Looking at *Cutoff* plot (Figure 12) we can see that Das_model_1 is however the best one from the local perspective. *3D plot*s indicate structural differences between all predictions. On the other hand, in Bujnicki_model_3 and Bujnicki_model_2 models, RNAlyzer points out local errors in several positions, which are however compensated on the global level by very accurate predictions in other regions (Figure 13).

**Figure 14.**
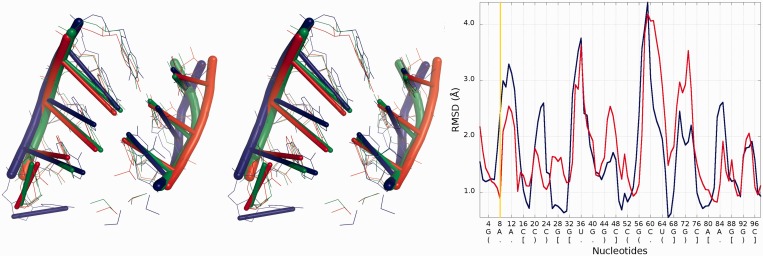
Superposition of Bujnicki_model_2 and Santalucia_model_1 (left) with reference structure; *Multi-model plot* (right) corresponds to discussed regions (green color—reference structure, red color—globally less accurate model (Santalucia_model_1), blue color—globally more accurate model (Bujnicki_model_2)).

Further analysis illustrates (Figure 14) that Bujnicki_model_1 in the local neighborhood located around nucleotide No 7 is actually worse than the globally less accurate Santalucia_model_1. The local RMSD between analyzed models is around 3 Å to the advantage of Santalucia_model_1, but globally the RMSD calculated for Bujnicki_model_1 is better by 1 Å than Santalucia_model_1.

Similar situation as presented above identified for Problem 1 is presented in Supplementary Figures S2 and S3.

### Problem 3 / Challenge case 3

**Figure 15.**
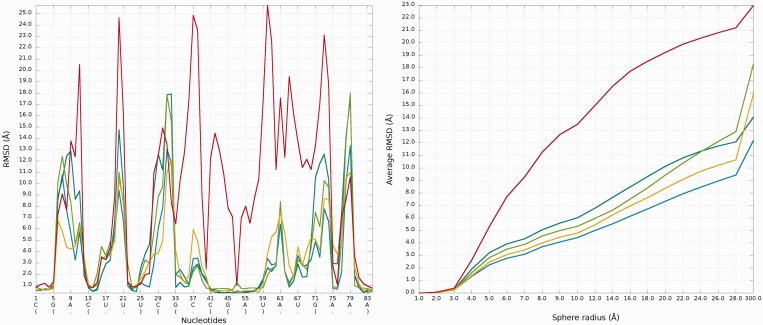
Problem_3—*Multi-model plot* (left)—prediction errors for all groups are indicated in several regions. *RMSD averaged plot* (right)—for low sphere radius values, Chen_model_1 is the best; different colors correspond to different models.

**Figure 16.**
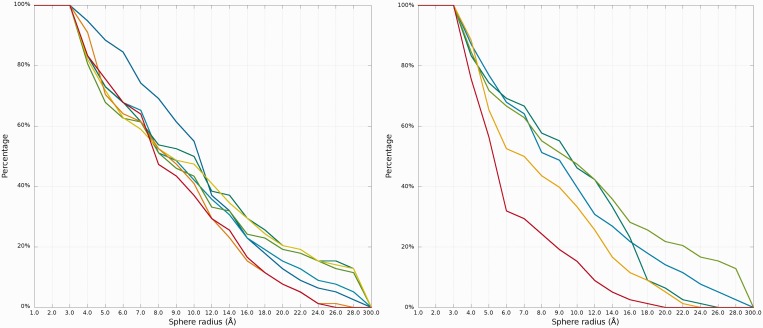
*Cutoff* plot illustrates outstanding local prediction of Chen_model_1 (precision 4 Å, sphere radius 6 Å); different colors correspond to different models.

The glycine riboswitch structure (28) is a relatively large RNA molecule and its prediction presented considerable challenge, compared with the other considered problems. Twelve models have been submitted for this target. Predicted models are relatively far from the reference structure, and the structural differences between the quality of submitted models are large (Figure 15). The analysis of the local neighborhood shows that there were submitted two models with similar prediction quality identified for spheres with radius up to 10 Å (Chen_model_1 and Dokholyan_model_1) (Figure 16). For a larger neighborhood (lower accuracy level), Chen_model_1 considerably surpassed all other models.

**Figure 17.**
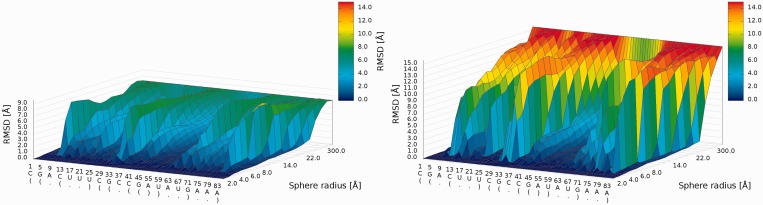
*3D plot* of Chen_model_1 (left) and Das_model_1 (right).

*3D plot*s show that Dokholyan_model_1 is worse than Chen_model_1 (Figure 17).

**Figure 18.**
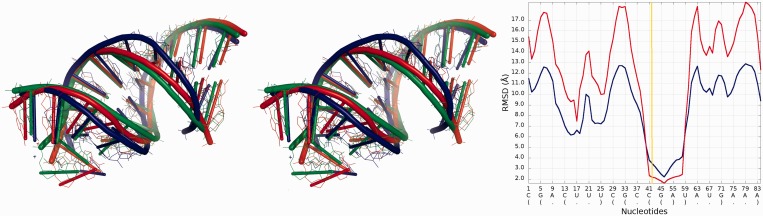
Superposition of Bujnicki_model_1 and Das_model_4 (left) with reference structure; *Multi-model plot* (right) corresponds to discussed regions (green color—reference structure, red color—globally less accurate model (Das_model_4), blue color—globally more accurate model (Bujnicki_model_1)).

Detailed analysis (Figure 18) illustrates that the RMSD computed for Bujnicki_model_1 in the local neighborhood around nucleotide No 42 is higher than for corresponding structural fragments identified in Das_model_3. The difference between the superimposed corresponding structural fragments is over 1 Å, but globally the RMSD for Bujnicki_model_1 is lower by 4 Å.

Similar situation as presented above identified for Problem 3 is presented in Supplementary Figure S4.

Validation of RNA tertiary structure models is a crucial issue in a structural biology. The presented method meets this challenge. RNAlyzer uses RMSD as the quality indicator for comparisons on all accuracy levels; however, any other metric can be used to calculate local structural distances between the extracted set of atoms. In the current version of the program, only RMSD is available, but other metrics will be added to that approach in the future. We showed that our method may be used to evaluate structures simultaneously at different levels of accuracy, to identify predictions that have locally correct tertiary structure even if they are less accurate globally, and vice versa. In the case of RNA structures, such comparison is important, as the *correctness* of prediction requires accurate modeling of both the details of local interactions and of the global molecule shape. In the case of modeling of functional noncoding RNA molecules such as riboswitches or ribozymes, it may be more important to model some fragments (e.g. binding/active sites) with very high accuracy, while the accuracy of other fragments and of the entire structure may be less important. On the other hand, in some other applications, the focus on the global structure may be more relevant. By analyzing models submitted to the RNA Puzzles challenge, we have demonstrated the utility of the proposed approach.

## SUPPLEMENTARY DATA

Supplementary Data are available at NAR Online: Supplementary Figures 1–4.

## FUNDING

EU Regional Development Fund [POIG.02.02.00-30-009/09]; European Regional Development Fund within Innovative Economy Programme [POIG.02.03.00-00-018/08 POWIEW]; National Science Center Poland [2012/06/A/ST6/00384 (to R.W.A.)]; National Science Center Poland [2012/05/B/ST6/03026]; Foundation for Polish Science [FNP, TEAM/2009-4/2 (to J.M.B.)]. Funding for open access charge: National Science Center Poland [2012/06/A/ST6/00384].

*Conflict of interest statement.* None declared.

## Supplementary Material

Supplementary Data
