# Supplementary material for: RNAlyzer—novel approach for quality analysis of RNA structural models
Source: Nucleic Acids Res. 2013 Apr 25;41(12):5978–90. doi: 10.1093/nar/gkt318 (PMC3695499; doi:10.1093/nar/gkt318)
Supplement: Supplementary Data [file supp_41_12_5978__index.html]

RNAlyzer—novel approach for quality analysis of RNA structural models — RNAlyzer—novel approach for quality analysis of RNA structural models — Supplementary Data 

# RNAlyzer—novel approach for quality analysis of RNA structural models

## Supplementary Data

files

**Files in this Data Supplement:**

- Supplementary Data - pdf file
