# Supplementary material for: RNAlyzer—novel approach for quality analysis of RNA structural models
Source: Nucleic Acids Res. 2013 Apr 25;41(12):5978–90. doi: 10.1093/nar/gkt318 (PMC3695499; doi:10.1093/nar/gkt318)
Supplement: Supplementary Data [file supp_gkt318_nar-02382-r-2012-File022.pdf]

SUPPLEMENTRY DATA

RNAlyzer – novel approach for quality analysis of RNA structural models

Piotr Łukasiak, Maciej Antczak, Tomasz Ratajczak, Janusz M. Bujnicki, Marta Szachniuk, Ryszard W. Adamiak, Mariusz Popenda, Jacek Błażewicz

Supplementary Data are available at NAR online:

Supplementary figures S1-S4

Problem 1 / Challenge case 1

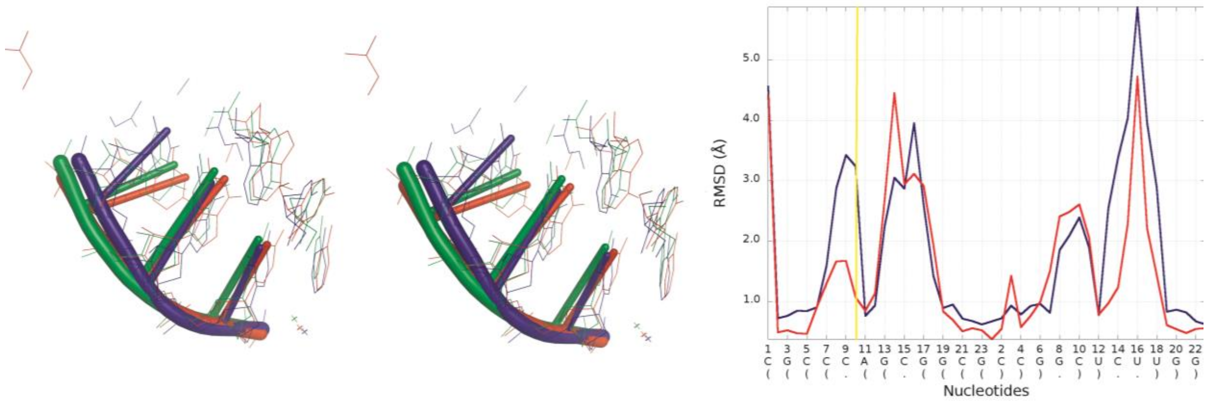

Figure S1. Superposition of Das_model_4 and Santalucia_model_1 models (left) with reference structure, *Multi-model plot* (right) corresponds to discussed regions (green color – reference structure, red color - globally less accurate model (Santalucia_model_1), blue color – globally more accurate model (Das_model_4)).

The structural comparison of Das_model_4 and Santalucia_model_1 models (Fig. S1) illustrates that the RMSD computed for local structural motive between models and the reference structure is around 2 Å (3,5 Å and 1,5 Å , respectively), but globally the RMSD computed for Das_model_4 is lower by 2 Å than RMSD calculated for Santalucia_model_1.

Problem 2 / Challenge case 2

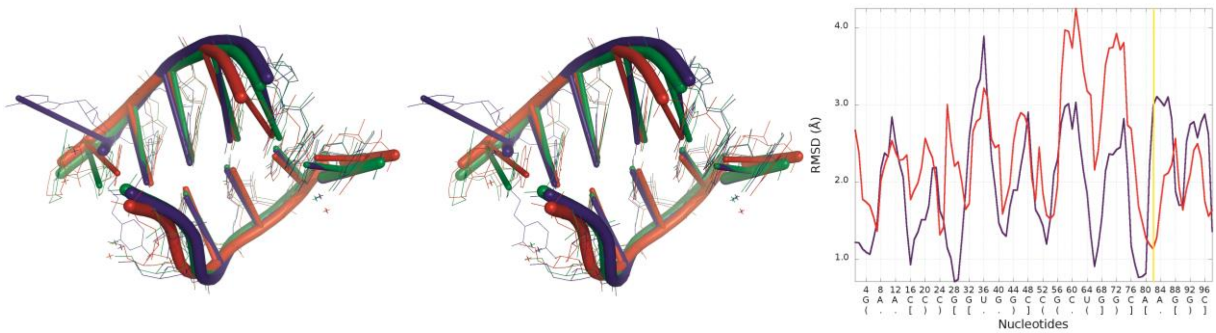

Figure S2. Superposition of Das_model_1 and Santalucia_model_1 models (left) with the reference structure, *Multi-model plot* (right) corresponds to discussed regions (green color – reference structure, red color - globally less accurate model (Santalucia_model_1), blue color – globally more accurate model (Das_model_1).

Detailed analysis (Fig. S2) shows that the RMSD computed for Das_model_1 in local neighborhood around nucleotide No 82 is higher than the RMSD calculated for Santalucia_model_1. The RMSD difference between local motive structures is around 1,5 Å, but globally RMSD for Das_model_1 is lower by 1 Å.

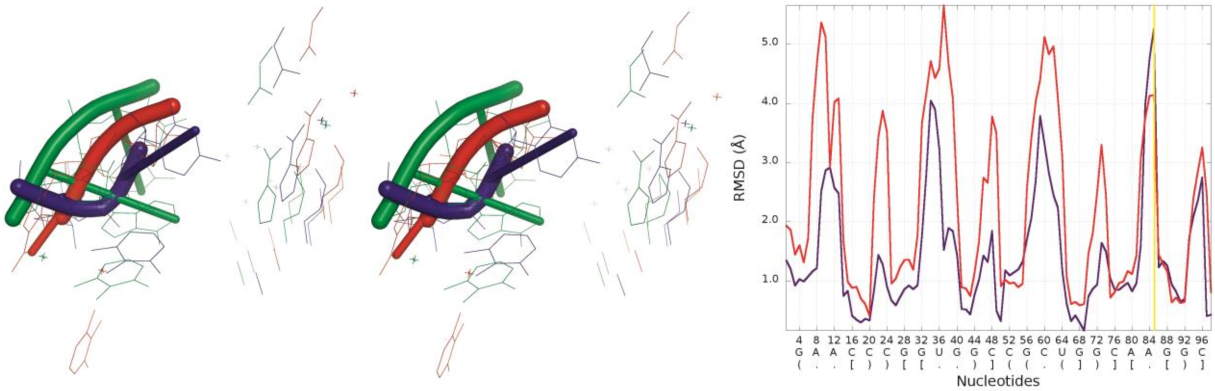

Figure S3. Superposition of Das_model_1 and Wildauer_model_1 (left) with reference structure, *Multi-model plot* (right) corresponds to discussed regions (green color – reference structure, red color - globally less accurate model (Wildauer_model_1), blue color – globally more accurate model (Das_model_1).

Detailed analysis (Fig. S3) shows that the RMSD computed for Das_model_1 in local neighborhood around nucleotide No 85 was is higher than RMSD calculated for Wildauer_model_1. The RMSD

difference between local motive structures is around 1 Å, but the globally RMSD calculated for Das_model_1 is lower by 1 Å.

Problem 3 / Challenge case 3

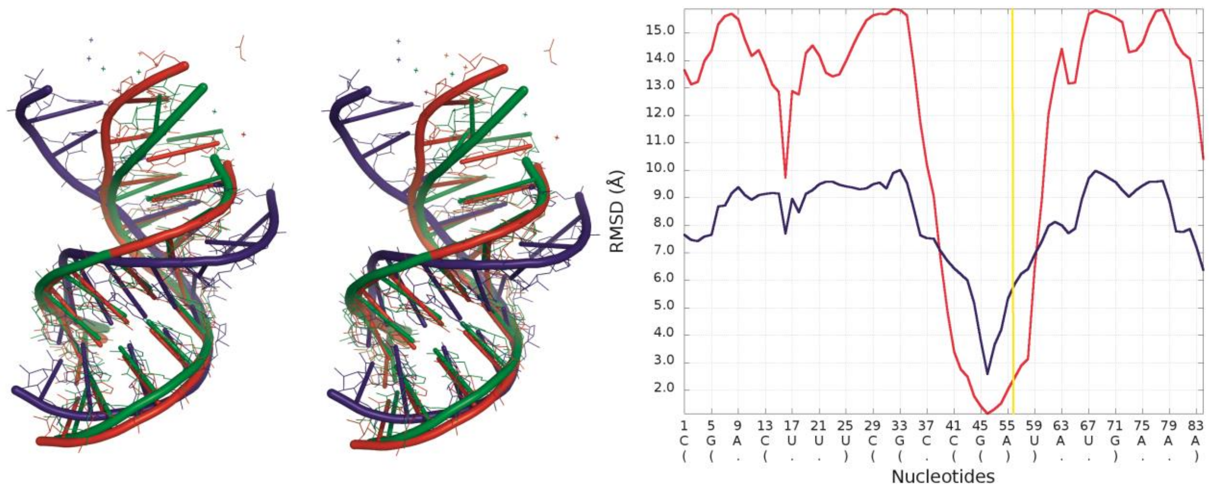

Figure S4. Superposition of Das_model_3 and Dokholyan_model_2 (left) with reference structure, *Multi-model plot* (right) corresponds to discussed regions (green color – reference structure, red color - globally less accurate model (Dokholyan_model_2), blue color – globally more accurate model (Das_model_3).

Detailed analysis (Fig. S4) shows that the RMSD computed for Das_model_3 in local neighborhood around nucleotide No 45 is higher than the RMSD calculated for Dokholyan_model_3. The RMSD difference between local motive structures is over 2 Å, but the globally RMSD for Das_model_3 is lower by 2 Å.
